# Bis(μ_4_-adamantane-1,3-di­carboxyl­ato-1κ*O* ^1^:2κ*O* ^1′^:3κ*O* ^3^:4κ*O* ^3′^)octa­carbonyl-1κ^2^ *C*,2κ^2^ *C*,3κ^2^ *C*,4κ^2^ *C*-tetra­kis­[tris­(4-methyl­phen­yl)phosphane]-1κ*P*,2κ*P*,3κ*P*,4κ*P*-tetra­osmium(I)(2 *Os*–*Os*)

**DOI:** 10.1107/S2414314620012043

**Published:** 2020-09-08

**Authors:** Diego F. Zometa Paniagua, Gregory L. Powell, Cynthia B. Powell, Eric W. Reinheimer

**Affiliations:** a Department of Chemistry & Biochemistry, Abilene Christian University, Abilene, Texas 79699-8132, USA; b Rigaku Americas Corp., 9009 New Trails Dr., The Woodlands, TX 77381, USA; Vienna University of Technology, Austria

**Keywords:** diosmium carbon­yl, sawhorse complex, supra­molecular loop, adamantane di­carboxyl­ate ligand, crystal structure

## Abstract

The title complex, [{Os_2_(CO)_4_(C_21_H_21_P)_2_}_2_(C_12_H_14_O_4_)_2_], is a centrosymmetric mol­ecular loop consisting of two Os—Os sawhorse units linked by two adamantane di­carboxyl­ato bridges. It was synthesized by the microwave-mediated reaction between Os_3_(CO)_12_ and adamantane-1,3-di­carb­oxy­lic acid. In contrast to the related complex [{Os_2_(CO)_6_}_2_(μ_4_-adamantane-1,3-di­acetate)_2_], the metal–metal axes within each mol­ecule are oriented parallel rather than perpendicular to one another. The crystal structure exhibits cavities that contain residual electron density peaks, but it was not possible to unambiguously identify the solvent therein. The contribution of the disordered solvent mol­ecules to the scattering was removed using the SQUEEZE (Spek (2015 ▸). *Acta Cryst*. C**71**, 9–18) routine in *PLATON* [Spek (2020 ▸). *Acta Cryst*. E**76**, 1–11]. These solvent mol­ecules are not considered in the given chemical formula and other crystal data.

## Abstract 2

[Os_2_(CO)_4_(tri-*p*-tolyl­phosphine)_2_]_2_(μ_4_-adamantane-1,3-di­carboxyl­ate)_2_ is a mol­ecular loop consisting of two parallel diosmium(I) sawhorse units linked together by two bridging di­carboxyl­ato ligands. The title compound is only the second example of a Group 8 dinuclear sawhorse complex with an adamantane-based di­carboxyl­ato ligand.

## Graphical abstract

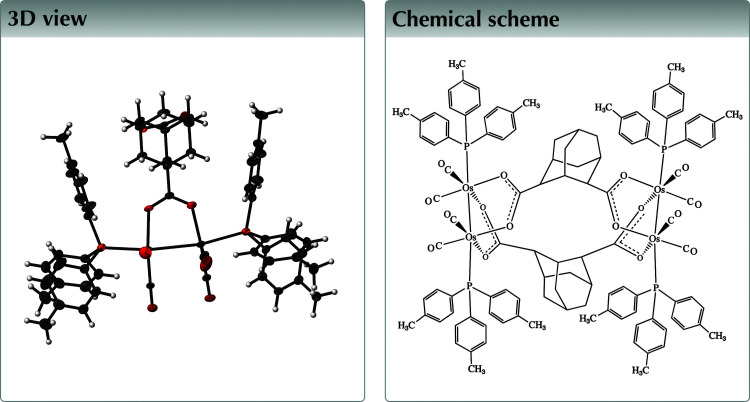

## Structure description

Group VIIIB sawhorse units with metal–metal bonds may have potential as building blocks for larger framework compounds including metal–organic frameworks (Köberl *et al.*, 2011 ▸; Therrien & Süss-Fink, 2009 ▸). There are nine Ru_2_ carboxyl­ato sawhorse assemblies in the Cambridge Structural Database (Version 5.41, last update November 2019; Groom *et al.*, 2016 ▸). Three are mol­ecular loops consisting of two sawhorse units (Bianchi *et al.*, 1981 ▸; Shiu *et al.*, 2002 ▸; Auzias *et al.*, 2007 ▸), five are mol­ecular triangles consisting of three sawhorse units (Auzias *et al.*, 2007 ▸; Süss-Fink *et al.*, 1990 ▸; Shiu *et al.*, 2003 ▸, 2010 ▸), and one is a mol­ecular square consisting of four sawhorse units (Shiu *et al.*, 2002 ▸). In all of these, the Ru—Ru axes are parallel rather than perpendicular to one another. The CSD also contains six Os_2_ carboxyl­ato sawhorse assemblies: five are mol­ecular loops of two sawhorse units and one is a mol­ecular triangle consisting of three sawhorse units (Fikes *et al.*, 2014 ▸; Gwini *et al.*, 2017 ▸). In all but one of these assemblies, the Os—Os axes within a mol­ecule are parallel to one another. Only the mol­ecular loop [Os_2_(CO)_6_]_2_(μ_4_-adamantane-1,3-di­acetate)_2_ has Os—Os axes that are oriented perpendicular to one another (Fikes *et al.*, 2014 ▸). No Ru_2_ sawhorse assemblies containing adamantane-based di­carboxyl­ato linkers have been reported. Our goal was to investigate the orientation of Os_2_ units that would result when using adamantane-1,3-di­carb­oxy­lic acid rather than adamantane-1,3-di­acetic acid as a starting material.

**Figure 1:**
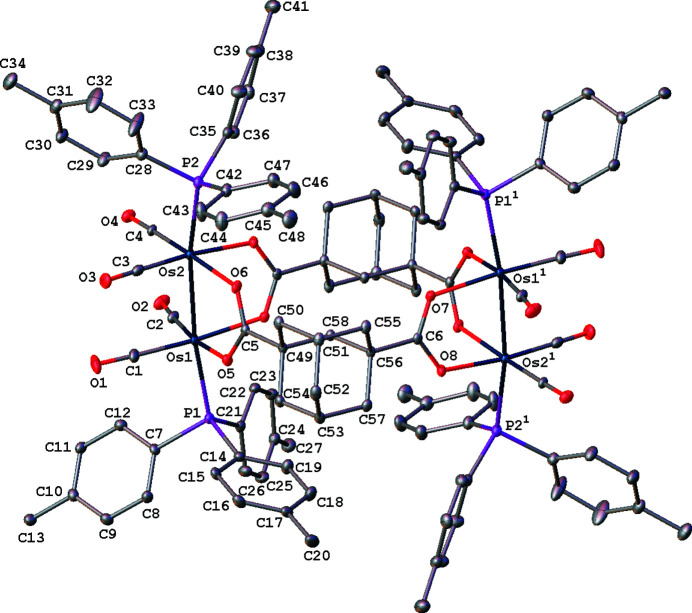
View of the title mol­ecule showing the atom-labeling scheme. Displacement ellipsoids are scaled to the 35% probability level. For the sake of clarity, all H atoms are omitted. [Symmetry code: (1) 1 − *x*, 1 − *y*, 1 – *z*].

The structure of the cluster mol­ecule in the title compound is illustrated in Fig. 1 ▸. The cluster entity resides on an inversion center and consists of a mol­ecular loop in which two Os_2_(CO)_4_(phosphine)_2_ sawhorse units are bridged by two adamantane-1,3-di­carboxyl­ato ligands. The four tri-*p*-tolyl­phosphine ligands occupy axial coordination sites with Os—Os—P angles of 170.20 (2) and 170.60 (2)°, which are typical for diosmium sawhorse complexes. Like Ru_2_ sawhorse carboxyl­ato macrocycles in which the Ru—Ru axes are parallel to one another, the two Os—Os axes in this structure are also parallel. This is in contrast to the related mol­ecular loop [Os_2_(CO)_6_]_2_(μ_4_-adamantane-1,3-di­acetate)_2_ in which the metal–metal axes within each mol­ecule are oriented perpendicular to one another (Fikes *et al.*, 2014 ▸). In the title compound, the Os—Os bond length is 2.7398 (2) Å. In [Os_2_(CO)_6_]_2_(μ_4_-adamantane-1,3-di­acetate)_2_, where the axial sites are occupied by carbonyl ligands instead of phosphine ligands, the metal–metal bond lengths are somewhat longer at 2.7433 (3) and 2.7561 (3) Å.

**Figure 2:**
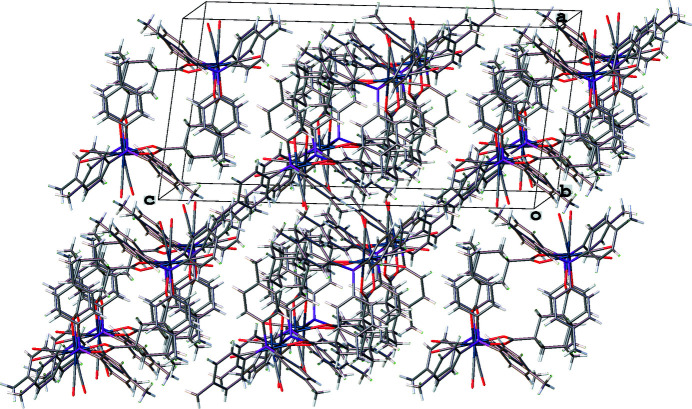
Packing of the title mol­ecules viewed approximately along the *b* axis.

**Figure 3:**
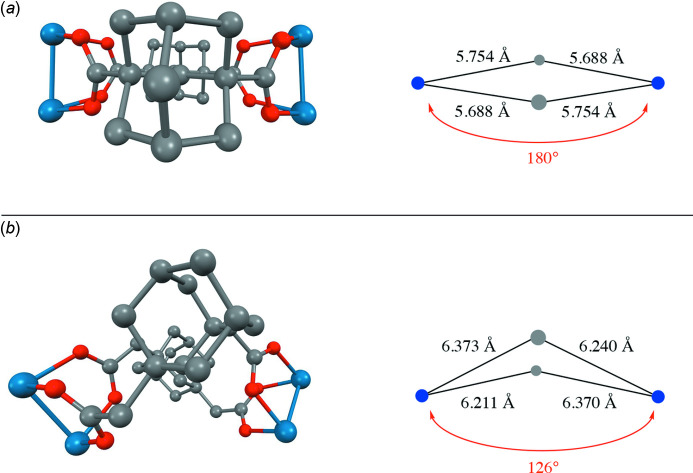
Views of the central shapes and core dimensions for (*a*) the title compound and (*b*) [Os_2_(CO)_6_]_2_(μ_4_-adamantane-1,3-di­acetate)_2_. On the left are perspective views in which atoms toward the front appear larger and atoms toward the back appear smaller. On the right are illustrations of the underlying core shapes in which blue dots represent the centroids of the two Os—Os units and gray dots represent the centroids of the two adamantane moieties.

The cluster mol­ecules of the title compound stack so that the Os—Os vectors are nearly parallel to the *b* axis and nearly perpendicular to the *a* axis. When viewed down the *b* axis, the central cavities of the mol­ecular loops align to form narrow channels, as shown in Fig. 2 ▸. Because sawhorse clusters with di­carboxyl­ato ligands have sometimes crystallized with solvent mol­ecules trapped in the center of the macrocycle, it is common to list the dimensions of the central cavity (Therrien & Süss-Fink, 2009 ▸). The cavity in the center of the title compound is a distorted rhombus with unique edge lengths of 4.684 (1) and 4.976 (1) Å as measured from the Os—Os midpoints to the central adamantane carbon atom C58. This cavity is smaller than that in [Os_2_(CO)_6_]_2_(μ_4_-adamantane-1,3-di­acetate)_2_ in which these distances average 5.2 Å (Fikes *et al.*, 2014 ▸). The size difference was expected since there are two fewer carbon atoms per linker ligand in the title compound. As a result of their small sizes, the centers of the mol­ecular loops cannot serve as a trap for solvent mol­ecules in either one of these complexes. As shown in Fig. 3 ▸, the central portions of these two mol­ecular loops also display different shapes. Connecting the centroids of the two Os—Os vectors and the centroids of the two adamantane groups leads to a butterfly shape in the case of [Os_2_(CO)_6_]_2_(μ_4_-adamantane-1,3-di­acetate)_2_ and an approximate square in the case of the title compound. The butterfly wings of the adamantane di­acetato complex are joined at an angle of 126°, while all four centroids are coplanar in the square of the title compound. The distances between adamantane centroids for the title cluster and for [Os_2_(CO)_6_]_2_(μ_4_-adamantane-1,3-di­acetate)_2_ are 7.087 (2) and 7.598 (2) Å, respectively. Despite the differences in dimensions and spacing for the adamantane-based ligands in these two complexes, the distances between Os—Os centroids are remarkably similar at 8.983 (2) and 8.964 (2) Å, respectively.

## Synthesis and crystallization

Os_3_(CO)_12_ (73.9 mg, 0.0815 mmol) and adamantane-1,3-di­carb­oxy­lic acid (29.2 mg, 0.130 mmol) were added to 7 ml of 1,2-di­chloro­benzene in a 35 ml microwave vessel. This solution was stirred and heated in the microwave reactor at 478 K for 13 minutes. The resulting solution had a pale-yellow color. The solvent was removed, then the residue was mixed with 25 ml of 1,2-di­chloro­ethane and 5 ml of aceto­nitrile and added to a 100 ml round-bottom flask equipped with a magnetic stir bar. Tri(*p*-tol­yl)phosphine (64.0 mg, 0.210 mmol) was added and the mixture was refluxed for 60 min. The solution was cooled to 277 K, 4 ml of *n*-hexane were added, and the products were isolated by fractional crystallization. The first fraction to precipitate was the desired product. Yield: 47.3 mg, 29.2%. IR (ν_CO_, cm^−1^): 2013 (*s*), 1967 (*w*), and 1938 (*s*). Analysis calculated (%) for C_116_H_112_O_16_Os_4_P_4_·C_6_H_14_: C 53.61, H 4.65; found: C 53.00, H 4.78. Crystals of the title compound were obtained by slow diffusion of hexa­nes into a di­chloro­methane solution.

## Refinement

Crystal data, data collection and structure refinement details are summarized in Table 1 ▸. Inter­stitial solvent mol­ecules could not be modeled in a satisfactory manner, so a solvent mask was generated revealing voids at (1/2, 0, 1/2) and (1/2, 1/2, 0), each with a volume of 394.4 Å^3^ and containing about 110 electrons. The contribution of the disordered solvent mol­ecules to the scattering was removed using the SQUEEZE (Spek, 2015 ▸) routine in *PLATON* (Spek, 2020 ▸). These solvent mol­ecules are not considered in the given chemical formula and other crystal data.

## Supplementary Material

Crystal structure: contains datablock(s) I. DOI: 10.1107/S2414314620012043/wm4137sup1.cif

Structure factors: contains datablock(s) I. DOI: 10.1107/S2414314620012043/wm4137Isup2.hkl

Supporting information file. DOI: 10.1107/S2414314620012043/wm4137Isup3.cdx

CCDC reference: 2026644

Additional supporting information:  crystallographic information; 3D view; checkCIF report

## Figures and Tables

**Table 1:** Experimental details

Crystal data	
Chemical formula	[Os_4_(C_12_H_14_O_4_)_2_(C_21_H_21_P)_4_(CO)_8_]
*M*_r_	2646.73
Crystal system, space group	Monoclinic, *P*2_1_/*c*
Temperature (K)	100
*a*, *b*, *c* (Å)	12.71423 (13), 17.04149 (18), 26.1784 (3)
β (°)	96.7054 (9)
*V* (Å^3^)	5633.26 (10)
*Z*	2
Radiation type	Cu *K*α
μ (mm^−1^)	9.33
Crystal size (mm)	0.27 × 0.21 × 0.10

Data collection	
Diffractometer	Rigaku SuperNova, Dual, Cu, Pilatus 200/300K
Absorption correction	Multi-scan (*CrysAlis PRO*; Rigaku OD, 2015 ▸)
*T*_min_, *T*_max_	0.411, 1.000
No. of measured, independent and observed [*I* > 2σ(*I*)] reflections	30975, 10059, 9674
*R*_int_	0.033
(sin θ/λ)_max_ (Å^−1^)	0.597

Refinement	
*R*[*F* ^2^ > 2σ(*F* ^2^)], *wR*(*F* ^2^), *S*	0.027, 0.070, 1.06
No. of reflections	10059
No. of parameters	637
H-atom treatment	H-atom parameters constrained
Δρ_max_, Δρ_min_ (e Å^−3^)	1.22, −1.34

Computer programs: *CrysAlis PRO* (Rigaku OD, 2015 ▸), *SHELXT2018/3* (Sheldrick, 2015*a* ▸), *SHELXL2014/7* (Sheldrick, 2015*b* ▸) and *OLEX2* (Dolomanov *et al.*, 2009 ▸).

## full crystallographic data

### Crystal data

**Table:**

[Os_4_(C_12_H_14_O_4_)_2_(C_21_H_21_P)_4_(CO)_8_]	*F*(000) = 2600
*M**_r_* = 2646.73	*D*_x_ = 1.560 Mg m^−^^3^
Monoclinic, *P*2_1_/*c*	Cu *K*α radiation, λ = 1.54184 Å
*a* = 12.71423 (13) Å	Cell parameters from 25347 reflections
*b* = 17.04149 (18) Å	θ = 2.6–77.3°
*c* = 26.1784 (3) Å	µ = 9.33 mm^−^^1^
β = 96.7054 (9)°	*T* = 100 K
*V* = 5633.26 (10) Å^3^	Block, yellow
*Z* = 2	0.27 × 0.21 × 0.10 mm

### Data collection

**Table:**

Rigaku SuperNova, Dual, Cu, Pilatus 200/300K diffractometer	10059 independent reflections
Radiation source: micro-focus sealed X-ray tube, SuperNova (Cu) X-ray Source	9674 reflections with *I* > 2σ(*I*)
Mirror monochromator	*R*_int_ = 0.033
ω scans	θ_max_ = 67.1°, θ_min_ = 5.7°
Absorption correction: multi-scan (CrysAlisPro; Rigaku OD, 2015)	*h* = −11→15
*T*_min_ = 0.411, *T*_max_ = 1.000	*k* = −20→20
30975 measured reflections	*l* = −31→31

### Refinement

**Table:**

Refinement on *F*^2^	0 restraints
Least-squares matrix: full	Hydrogen site location: inferred from neighbouring sites
*R*[*F*^2^ > 2σ(*F*^2^)] = 0.027	H-atom parameters constrained
*wR*(*F*^2^) = 0.070	*w* = 1/[σ^2^(*F*_o_^2^) + (0.0375*P*)^2^ + 7.1306*P*] where *P* = (*F*_o_^2^ + 2*F*_c_^2^)/3
*S* = 1.06	(Δ/σ)_max_ = 0.003
10059 reflections	Δρ_max_ = 1.22 e Å^−^^3^
637 parameters	Δρ_min_ = −1.34 e Å^−^^3^

### Special details

**Table:**

Geometry. All esds (except the esd in the dihedral angle between two l.s. planes) are estimated using the full covariance matrix. The cell esds are taken into account individually in the estimation of esds in distances, angles and torsion angles; correlations between esds in cell parameters are only used when they are defined by crystal symmetry. An approximate (isotropic) treatment of cell esds is used for estimating esds involving l.s. planes.

### Fractional atomic coordinates and isotropic or equivalent isotropic displacement parameters (Å^2^)

**Table:**

	*x*	*y*	*z*	*U*_iso_*/*U*_eq_	
Os2	0.25720 (2)	0.61407 (2)	0.60158 (2)	0.01693 (5)	
Os1	0.24924 (2)	0.45454 (2)	0.61351 (2)	0.01735 (5)	
P2	0.29032 (6)	0.74958 (5)	0.58494 (3)	0.02141 (17)	
P1	0.27376 (6)	0.31514 (5)	0.62196 (3)	0.02018 (17)	
O5	0.41485 (18)	0.46765 (13)	0.63120 (9)	0.0219 (5)	
O7	0.70994 (18)	0.54451 (13)	0.46337 (9)	0.0200 (5)	
O8	0.73816 (17)	0.41608 (13)	0.47687 (8)	0.0211 (5)	
O6	0.42202 (16)	0.59267 (14)	0.60497 (9)	0.0217 (5)	
O4	0.02026 (18)	0.62535 (14)	0.59194 (10)	0.0296 (6)	
O3	0.2741 (2)	0.64628 (16)	0.71537 (9)	0.0341 (6)	
O2	0.0191 (2)	0.44367 (16)	0.57153 (12)	0.0384 (7)	
O1	0.1945 (3)	0.46999 (17)	0.72174 (11)	0.0466 (8)	
C6	0.7201 (2)	0.48462 (19)	0.49227 (12)	0.0178 (6)	
C49	0.5819 (2)	0.51894 (19)	0.61418 (12)	0.0184 (6)	
C51	0.7592 (2)	0.5842 (2)	0.62477 (13)	0.0243 (7)	
H51	0.7988	0.6327	0.6368	0.029*	
C50	0.6422 (2)	0.5942 (2)	0.63210 (12)	0.0219 (7)	
H50A	0.6121	0.6396	0.6118	0.026*	
H50B	0.6351	0.6041	0.6688	0.026*	
C28	0.2264 (3)	0.8181 (2)	0.62644 (13)	0.0253 (7)	
C54	0.6286 (3)	0.4485 (2)	0.64515 (13)	0.0231 (7)	
H54A	0.5895	0.4003	0.6334	0.028*	
H54B	0.6212	0.4562	0.6820	0.028*	
C4	0.1111 (2)	0.62182 (18)	0.59547 (13)	0.0195 (7)	
C58	0.5922 (2)	0.50562 (19)	0.55659 (11)	0.0176 (6)	
H58A	0.5522	0.4581	0.5443	0.021*	
H58B	0.5621	0.5510	0.5363	0.021*	
C8	0.2435 (3)	0.2145 (2)	0.70621 (13)	0.0267 (7)	
H8	0.3141	0.1970	0.7052	0.032*	
C1	0.2165 (3)	0.4633 (2)	0.68037 (15)	0.0286 (8)	
C56	0.7100 (2)	0.49564 (19)	0.54905 (12)	0.0184 (6)	
C53	0.7461 (3)	0.4393 (2)	0.63792 (13)	0.0243 (7)	
H53	0.7765	0.3936	0.6585	0.029*	
C5	0.4636 (2)	0.52736 (19)	0.61791 (12)	0.0180 (6)	
C36	0.1640 (3)	0.7508 (2)	0.49043 (14)	0.0287 (8)	
H36	0.1456	0.6987	0.4988	0.034*	
C2	0.1064 (3)	0.4478 (2)	0.58887 (15)	0.0249 (7)	
C29	0.1324 (3)	0.7978 (2)	0.64379 (16)	0.0339 (9)	
H29	0.1044	0.7468	0.6366	0.041*	
C42	0.4299 (3)	0.7757 (2)	0.59678 (14)	0.0257 (7)	
C55	0.7692 (2)	0.5714 (2)	0.56791 (12)	0.0212 (7)	
H55A	0.8449	0.5670	0.5628	0.025*	
H55B	0.7388	0.6168	0.5477	0.025*	
C10	0.0774 (3)	0.2041 (2)	0.74291 (13)	0.0251 (7)	
C16	0.5683 (3)	0.2778 (2)	0.69879 (14)	0.0333 (8)	
H16	0.6021	0.2862	0.7327	0.040*	
C52	0.8063 (3)	0.5139 (2)	0.65596 (13)	0.0283 (8)	
H52A	0.8820	0.5081	0.6513	0.034*	
H52B	0.8010	0.5226	0.6930	0.034*	
C47	0.4942 (3)	0.7828 (2)	0.55779 (15)	0.0351 (9)	
H47	0.4646	0.7769	0.5230	0.042*	
C7	0.2028 (3)	0.2735 (2)	0.67298 (13)	0.0224 (7)	
C25	0.1718 (3)	0.1197 (2)	0.53986 (15)	0.0318 (8)	
H25	0.1765	0.0645	0.5449	0.038*	
C9	0.1820 (3)	0.1808 (2)	0.74093 (14)	0.0296 (8)	
H9	0.2118	0.1412	0.7637	0.036*	
C12	0.0982 (3)	0.2973 (2)	0.67532 (14)	0.0266 (7)	
H12	0.0685	0.3375	0.6530	0.032*	
C3	0.2664 (3)	0.6339 (2)	0.67137 (15)	0.0256 (7)	
C22	0.1644 (3)	0.2799 (2)	0.52520 (13)	0.0242 (7)	
H22	0.1625	0.3349	0.5195	0.029*	
C30	0.0769 (3)	0.8496 (2)	0.67153 (16)	0.0356 (9)	
H30	0.0121	0.8330	0.6828	0.043*	
C35	0.2418 (3)	0.7906 (2)	0.52221 (13)	0.0256 (7)	
C44	0.5840 (3)	0.7957 (3)	0.65881 (16)	0.0401 (10)	
H44	0.6140	0.7988	0.6937	0.048*	
C57	0.7557 (2)	0.4251 (2)	0.58085 (12)	0.0213 (7)	
H57A	0.7167	0.3769	0.5692	0.026*	
H57B	0.8311	0.4178	0.5758	0.026*	
C14	0.4102 (3)	0.2842 (2)	0.63794 (13)	0.0231 (7)	
C37	0.1129 (3)	0.7861 (2)	0.44668 (14)	0.0327 (8)	
H37	0.0594	0.7581	0.4258	0.039*	
C21	0.2208 (3)	0.2501 (2)	0.56945 (13)	0.0234 (7)	
C11	0.0376 (3)	0.2631 (2)	0.70974 (14)	0.0260 (7)	
H11	−0.0331	0.2805	0.7107	0.031*	
C13	0.0095 (3)	0.1657 (2)	0.77931 (15)	0.0317 (8)	
H13A	0.0155	0.1085	0.7767	0.048*	
H13B	−0.0645	0.1813	0.7703	0.048*	
H13C	0.0335	0.1822	0.8146	0.048*	
C24	0.1110 (3)	0.1492 (2)	0.49648 (14)	0.0277 (7)	
C15	0.4625 (3)	0.2981 (2)	0.68698 (13)	0.0268 (7)	
H15	0.4254	0.3217	0.7125	0.032*	
C27	0.0453 (3)	0.0956 (2)	0.46000 (16)	0.0348 (8)	
H27A	0.0649	0.0409	0.4680	0.052*	
H27B	0.0579	0.1076	0.4246	0.052*	
H27C	−0.0299	0.1033	0.4636	0.052*	
C19	0.4682 (3)	0.2526 (2)	0.60059 (15)	0.0329 (8)	
H19	0.4345	0.2438	0.5667	0.039*	
C17	0.6259 (3)	0.2454 (2)	0.66182 (15)	0.0316 (8)	
C40	0.2691 (3)	0.8659 (2)	0.50738 (16)	0.0348 (9)	
H40	0.3234	0.8937	0.5279	0.042*	
C45	0.6480 (3)	0.8063 (2)	0.62011 (16)	0.0334 (8)	
C23	0.1108 (3)	0.2303 (2)	0.48932 (13)	0.0253 (7)	
H23	0.0730	0.2520	0.4591	0.030*	
C46	0.6026 (3)	0.7985 (3)	0.56931 (16)	0.0396 (9)	
H46	0.6456	0.8039	0.5422	0.048*	
C43	0.4768 (3)	0.7806 (3)	0.64735 (15)	0.0397 (10)	
H43	0.4345	0.7735	0.6746	0.048*	
C20	0.7412 (3)	0.2254 (3)	0.67495 (17)	0.0424 (10)	
H20A	0.7688	0.2518	0.7070	0.064*	
H20B	0.7809	0.2429	0.6471	0.064*	
H20C	0.7490	0.1685	0.6793	0.064*	
C41	0.0788 (4)	0.9010 (3)	0.38623 (17)	0.0426 (10)	
H41A	0.0493	0.8607	0.3619	0.064*	
H41B	0.1272	0.9347	0.3696	0.064*	
H41C	0.0211	0.9328	0.3970	0.064*	
C26	0.2254 (3)	0.1689 (2)	0.57566 (14)	0.0306 (8)	
H26	0.2660	0.1472	0.6050	0.037*	
C18	0.5747 (3)	0.2341 (2)	0.61253 (16)	0.0358 (9)	
H18	0.6132	0.2133	0.5866	0.043*	
C39	0.2188 (3)	0.9006 (2)	0.46349 (17)	0.0389 (9)	
H39	0.2394	0.9518	0.4542	0.047*	
C31	0.1127 (3)	0.9229 (2)	0.68301 (14)	0.0319 (8)	
C38	0.1385 (3)	0.8618 (2)	0.43278 (15)	0.0327 (8)	
C48	0.7645 (3)	0.8245 (3)	0.63302 (19)	0.0437 (10)	
H48A	0.7767	0.8804	0.6274	0.066*	
H48B	0.8056	0.7933	0.6109	0.066*	
H48C	0.7867	0.8114	0.6691	0.066*	
C33	0.2633 (4)	0.8923 (3)	0.6380 (3)	0.0705 (19)	
H33	0.3290	0.9083	0.6274	0.085*	
C34	0.0505 (3)	0.9778 (3)	0.71285 (18)	0.0422 (10)	
H34A	−0.0248	0.9742	0.6998	0.063*	
H34B	0.0752	1.0317	0.7089	0.063*	
H34C	0.0605	0.9632	0.7493	0.063*	
C32	0.2065 (5)	0.9446 (3)	0.6649 (3)	0.076 (2)	
H32	0.2327	0.9963	0.6710	0.091*	

### Atomic displacement parameters (Å^2^)

**Table:**

	*U*^11^	*U*^22^	*U*^33^	*U*^12^	*U*^13^	*U*^23^
Os2	0.01309 (8)	0.01962 (9)	0.01877 (8)	−0.00181 (5)	0.00478 (5)	−0.00414 (5)
Os1	0.01328 (8)	0.02051 (8)	0.01932 (8)	−0.00221 (5)	0.00641 (5)	−0.00143 (5)
P2	0.0186 (4)	0.0222 (4)	0.0242 (4)	−0.0045 (3)	0.0059 (3)	−0.0036 (3)
P1	0.0172 (4)	0.0220 (4)	0.0222 (4)	0.0000 (3)	0.0059 (3)	0.0006 (3)
O5	0.0147 (11)	0.0282 (13)	0.0231 (12)	−0.0018 (9)	0.0034 (9)	0.0019 (9)
O7	0.0165 (11)	0.0224 (12)	0.0214 (11)	0.0011 (8)	0.0034 (9)	−0.0022 (9)
O8	0.0226 (11)	0.0217 (12)	0.0205 (11)	−0.0022 (9)	0.0084 (9)	−0.0025 (9)
O6	0.0114 (10)	0.0246 (12)	0.0298 (12)	−0.0033 (9)	0.0058 (9)	−0.0060 (10)
O4	0.0179 (13)	0.0280 (13)	0.0428 (15)	0.0003 (10)	0.0025 (10)	−0.0016 (11)
O3	0.0432 (15)	0.0413 (16)	0.0176 (13)	0.0019 (12)	0.0031 (10)	−0.0092 (11)
O2	0.0154 (14)	0.0328 (14)	0.067 (2)	−0.0014 (11)	0.0043 (12)	−0.0038 (13)
O1	0.068 (2)	0.0454 (18)	0.0316 (15)	−0.0182 (14)	0.0294 (15)	−0.0102 (13)
C6	0.0091 (14)	0.0246 (17)	0.0200 (15)	−0.0028 (12)	0.0025 (11)	−0.0029 (13)
C49	0.0145 (15)	0.0237 (16)	0.0173 (15)	−0.0032 (13)	0.0032 (11)	−0.0023 (13)
C51	0.0166 (16)	0.0322 (19)	0.0237 (17)	−0.0056 (14)	0.0010 (12)	−0.0089 (15)
C50	0.0179 (16)	0.0292 (18)	0.0185 (15)	−0.0041 (13)	0.0019 (12)	−0.0074 (14)
C28	0.0244 (17)	0.0239 (18)	0.0279 (17)	−0.0025 (14)	0.0046 (14)	−0.0055 (14)
C54	0.0218 (17)	0.0294 (18)	0.0183 (15)	0.0000 (14)	0.0033 (13)	0.0002 (13)
C4	0.0126 (16)	0.0195 (16)	0.0275 (17)	0.0003 (12)	0.0071 (12)	−0.0022 (13)
C58	0.0139 (15)	0.0222 (16)	0.0165 (15)	−0.0015 (12)	0.0013 (11)	−0.0027 (12)
C8	0.0204 (17)	0.032 (2)	0.0280 (18)	0.0026 (14)	0.0052 (13)	0.0035 (15)
C1	0.027 (2)	0.0269 (19)	0.034 (2)	−0.0057 (14)	0.0150 (16)	−0.0028 (15)
C56	0.0123 (14)	0.0248 (17)	0.0183 (15)	−0.0017 (13)	0.0022 (11)	−0.0038 (13)
C53	0.0190 (17)	0.0348 (19)	0.0189 (16)	0.0041 (14)	0.0008 (12)	0.0031 (14)
C5	0.0140 (15)	0.0249 (17)	0.0153 (14)	−0.0017 (12)	0.0020 (11)	−0.0039 (12)
C36	0.0328 (19)	0.0258 (18)	0.0284 (18)	−0.0039 (15)	0.0068 (14)	−0.0032 (15)
C2	0.0155 (18)	0.0221 (17)	0.039 (2)	−0.0012 (13)	0.0092 (14)	−0.0005 (15)
C29	0.0299 (19)	0.0235 (18)	0.051 (2)	−0.0059 (15)	0.0149 (17)	−0.0109 (17)
C42	0.0217 (17)	0.0261 (18)	0.0306 (18)	−0.0049 (14)	0.0082 (14)	−0.0056 (14)
C55	0.0132 (15)	0.0274 (18)	0.0232 (16)	−0.0066 (13)	0.0028 (12)	−0.0073 (14)
C10	0.0251 (17)	0.0256 (17)	0.0253 (17)	−0.0045 (14)	0.0063 (13)	−0.0019 (14)
C16	0.0259 (18)	0.046 (2)	0.0278 (18)	−0.0002 (16)	0.0027 (14)	0.0137 (17)
C52	0.0163 (16)	0.046 (2)	0.0220 (17)	−0.0009 (15)	−0.0013 (13)	−0.0053 (16)
C47	0.0267 (19)	0.046 (2)	0.034 (2)	−0.0009 (17)	0.0079 (15)	0.0026 (17)
C7	0.0209 (16)	0.0224 (17)	0.0249 (16)	−0.0044 (13)	0.0075 (13)	0.0022 (13)
C25	0.038 (2)	0.0196 (18)	0.039 (2)	0.0009 (15)	0.0068 (17)	0.0000 (15)
C9	0.0272 (18)	0.031 (2)	0.0299 (18)	−0.0001 (15)	0.0024 (14)	0.0080 (15)
C12	0.0238 (17)	0.0259 (18)	0.0302 (18)	0.0001 (14)	0.0027 (14)	0.0033 (15)
C3	0.0173 (16)	0.0211 (17)	0.039 (2)	0.0014 (13)	0.0054 (14)	−0.0002 (15)
C22	0.0245 (17)	0.0196 (16)	0.0295 (17)	0.0024 (13)	0.0078 (13)	0.0026 (14)
C30	0.0288 (19)	0.029 (2)	0.052 (2)	−0.0015 (16)	0.0183 (17)	−0.0027 (18)
C35	0.0224 (17)	0.0265 (18)	0.0296 (18)	0.0018 (14)	0.0099 (14)	−0.0003 (14)
C44	0.0270 (19)	0.059 (3)	0.035 (2)	−0.0132 (18)	0.0044 (16)	−0.0151 (19)
C57	0.0128 (15)	0.0266 (18)	0.0243 (17)	−0.0007 (13)	0.0017 (12)	−0.0029 (14)
C14	0.0216 (16)	0.0226 (17)	0.0256 (17)	0.0020 (13)	0.0051 (13)	0.0057 (14)
C37	0.035 (2)	0.039 (2)	0.0240 (18)	−0.0037 (17)	0.0035 (15)	−0.0061 (16)
C21	0.0181 (16)	0.0254 (17)	0.0280 (17)	0.0019 (13)	0.0086 (13)	0.0017 (14)
C11	0.0181 (16)	0.0250 (18)	0.0363 (19)	0.0000 (13)	0.0089 (14)	0.0007 (15)
C13	0.0289 (19)	0.0284 (19)	0.040 (2)	0.0021 (15)	0.0118 (16)	0.0084 (16)
C24	0.0261 (17)	0.0286 (19)	0.0294 (18)	−0.0009 (15)	0.0071 (14)	−0.0024 (15)
C15	0.0219 (17)	0.0334 (19)	0.0266 (17)	−0.0012 (14)	0.0095 (13)	0.0066 (15)
C27	0.037 (2)	0.0273 (19)	0.040 (2)	−0.0048 (16)	0.0055 (17)	−0.0051 (17)
C19	0.0241 (18)	0.042 (2)	0.0326 (19)	0.0046 (16)	0.0052 (15)	−0.0028 (17)
C17	0.0240 (18)	0.034 (2)	0.038 (2)	0.0064 (15)	0.0113 (15)	0.0152 (17)
C40	0.0285 (19)	0.0262 (19)	0.049 (2)	−0.0065 (16)	−0.0002 (17)	0.0035 (17)
C45	0.0255 (18)	0.034 (2)	0.041 (2)	−0.0063 (16)	0.0047 (15)	−0.0042 (17)
C23	0.0234 (17)	0.0245 (18)	0.0276 (17)	0.0010 (14)	0.0014 (13)	0.0006 (14)
C46	0.0238 (19)	0.057 (3)	0.040 (2)	−0.0041 (18)	0.0115 (16)	0.008 (2)
C43	0.0272 (19)	0.062 (3)	0.031 (2)	−0.0157 (19)	0.0108 (16)	−0.0126 (19)
C20	0.027 (2)	0.053 (3)	0.050 (2)	0.0137 (18)	0.0124 (17)	0.023 (2)
C41	0.052 (3)	0.037 (2)	0.038 (2)	0.0079 (19)	0.0030 (19)	0.0016 (18)
C26	0.036 (2)	0.0274 (19)	0.0283 (18)	0.0040 (15)	0.0015 (15)	0.0041 (15)
C18	0.0268 (19)	0.041 (2)	0.042 (2)	0.0074 (16)	0.0121 (16)	−0.0013 (18)
C39	0.039 (2)	0.028 (2)	0.049 (2)	−0.0021 (17)	0.0030 (18)	0.0074 (18)
C31	0.0299 (19)	0.034 (2)	0.0311 (19)	0.0033 (16)	0.0013 (15)	−0.0105 (16)
C38	0.035 (2)	0.033 (2)	0.0301 (19)	0.0071 (16)	0.0070 (15)	0.0001 (16)
C48	0.0242 (19)	0.050 (3)	0.058 (3)	−0.0117 (18)	0.0101 (18)	−0.008 (2)
C33	0.050 (3)	0.044 (3)	0.128 (5)	−0.023 (2)	0.054 (3)	−0.044 (3)
C34	0.039 (2)	0.041 (2)	0.047 (2)	0.0045 (18)	0.0052 (19)	−0.019 (2)
C32	0.071 (4)	0.043 (3)	0.123 (5)	−0.026 (3)	0.051 (4)	−0.050 (3)

### Geometric parameters (Å, º)

**Table:**

Os2—Os1	2.7398 (2)	C52—H52B	0.9900
Os2—P2	2.3964 (9)	C47—H47	0.9500
Os2—O8^i^	2.124 (2)	C47—C46	1.401 (5)
Os2—O6	2.119 (2)	C7—C12	1.398 (5)
Os2—C4	1.850 (3)	C25—H25	0.9500
Os2—C3	1.848 (4)	C25—C24	1.391 (5)
Os1—P1	2.4028 (9)	C25—C26	1.377 (5)
Os1—O5	2.114 (2)	C9—H9	0.9500
Os1—O7^i^	2.136 (2)	C12—H12	0.9500
Os1—C1	1.852 (4)	C12—C11	1.381 (5)
Os1—C2	1.858 (4)	C22—H22	0.9500
P2—C28	1.846 (3)	C22—C21	1.386 (5)
P2—C42	1.821 (3)	C22—C23	1.382 (5)
P2—C35	1.825 (4)	C30—H30	0.9500
P1—C7	1.840 (3)	C30—C31	1.353 (5)
P1—C14	1.815 (3)	C35—C40	1.396 (5)
P1—C21	1.832 (4)	C44—H44	0.9500
O5—C5	1.261 (4)	C44—C45	1.383 (6)
O7—Os1^i^	2.136 (2)	C44—C43	1.385 (5)
O7—C6	1.268 (4)	C57—H57A	0.9900
O8—Os2^i^	2.124 (2)	C57—H57B	0.9900
O8—C6	1.265 (4)	C14—C15	1.395 (5)
O6—C5	1.261 (4)	C14—C19	1.399 (5)
O4—C4	1.150 (4)	C37—H37	0.9500
O3—C3	1.164 (4)	C37—C38	1.389 (6)
O2—C2	1.152 (4)	C21—C26	1.393 (5)
O1—C1	1.155 (5)	C11—H11	0.9500
C6—C56	1.518 (4)	C13—H13A	0.9800
C49—C50	1.539 (4)	C13—H13B	0.9800
C49—C54	1.530 (5)	C13—H13C	0.9800
C49—C58	1.545 (4)	C24—C27	1.503 (5)
C49—C5	1.525 (4)	C24—C23	1.396 (5)
C51—H51	1.0000	C15—H15	0.9500
C51—C50	1.532 (4)	C27—H27A	0.9800
C51—C55	1.524 (5)	C27—H27B	0.9800
C51—C52	1.531 (5)	C27—H27C	0.9800
C50—H50A	0.9900	C19—H19	0.9500
C50—H50B	0.9900	C19—C18	1.390 (5)
C28—C29	1.371 (5)	C17—C20	1.506 (5)
C28—C33	1.371 (5)	C17—C18	1.389 (6)
C54—H54A	0.9900	C40—H40	0.9500
C54—H54B	0.9900	C40—C39	1.382 (6)
C54—C53	1.536 (5)	C45—C46	1.393 (6)
C58—H58A	0.9900	C45—C48	1.513 (5)
C58—H58B	0.9900	C23—H23	0.9500
C58—C56	1.543 (4)	C46—H46	0.9500
C8—H8	0.9500	C43—H43	0.9500
C8—C7	1.389 (5)	C20—H20A	0.9800
C8—C9	1.390 (5)	C20—H20B	0.9800
C56—C55	1.546 (4)	C20—H20C	0.9800
C56—C57	1.537 (5)	C41—H41A	0.9800
C53—H53	1.0000	C41—H41B	0.9800
C53—C52	1.530 (5)	C41—H41C	0.9800
C53—C57	1.532 (4)	C41—C38	1.513 (5)
C36—H36	0.9500	C26—H26	0.9500
C36—C35	1.392 (5)	C18—H18	0.9500
C36—C37	1.387 (5)	C39—H39	0.9500
C29—H29	0.9500	C39—C38	1.391 (6)
C29—C30	1.386 (5)	C31—C34	1.501 (5)
C42—C47	1.386 (5)	C31—C32	1.384 (7)
C42—C43	1.389 (5)	C48—H48A	0.9800
C55—H55A	0.9900	C48—H48B	0.9800
C55—H55B	0.9900	C48—H48C	0.9800
C10—C9	1.395 (5)	C33—H33	0.9500
C10—C11	1.385 (5)	C33—C32	1.389 (7)
C10—C13	1.507 (5)	C34—H34A	0.9800
C16—H16	0.9500	C34—H34B	0.9800
C16—C15	1.389 (5)	C34—H34C	0.9800
C16—C17	1.394 (5)	C32—H32	0.9500
C52—H52A	0.9900		
			
P2—Os2—Os1	170.60 (2)	C42—C47—H47	119.7
O8^i^—Os2—Os1	82.86 (6)	C42—C47—C46	120.5 (4)
O8^i^—Os2—P2	91.84 (6)	C46—C47—H47	119.7
O6—Os2—Os1	82.76 (6)	C8—C7—P1	123.7 (3)
O6—Os2—P2	88.83 (7)	C8—C7—C12	118.0 (3)
O6—Os2—O8^i^	81.95 (9)	C12—C7—P1	118.0 (3)
C4—Os2—Os1	91.75 (10)	C24—C25—H25	119.4
C4—Os2—P2	96.40 (10)	C26—C25—H25	119.4
C4—Os2—O8^i^	94.28 (12)	C26—C25—C24	121.3 (3)
C4—Os2—O6	173.67 (11)	C8—C9—C10	121.2 (3)
C3—Os2—Os1	93.86 (11)	C8—C9—H9	119.4
C3—Os2—P2	90.67 (11)	C10—C9—H9	119.4
C3—Os2—O8^i^	173.78 (12)	C7—C12—H12	119.6
C3—Os2—O6	92.41 (12)	C11—C12—C7	120.8 (3)
C3—Os2—C4	91.09 (15)	C11—C12—H12	119.6
P1—Os1—Os2	170.20 (2)	O3—C3—Os2	178.8 (3)
O5—Os1—Os2	82.59 (6)	C21—C22—H22	119.7
O5—Os1—P1	88.23 (6)	C23—C22—H22	119.7
O5—Os1—O7^i^	81.97 (9)	C23—C22—C21	120.7 (3)
O7^i^—Os1—Os2	82.46 (6)	C29—C30—H30	119.1
O7^i^—Os1—P1	92.92 (6)	C31—C30—C29	121.8 (3)
C1—Os1—Os2	92.49 (11)	C31—C30—H30	119.1
C1—Os1—P1	91.92 (11)	C36—C35—P2	119.6 (3)
C1—Os1—O5	96.48 (14)	C36—C35—C40	117.7 (3)
C1—Os1—O7^i^	174.86 (12)	C40—C35—P2	122.2 (3)
C1—Os1—C2	90.83 (17)	C45—C44—H44	119.6
C2—Os1—Os2	94.00 (11)	C45—C44—C43	120.9 (4)
C2—Os1—P1	94.68 (11)	C43—C44—H44	119.6
C2—Os1—O5	172.05 (13)	C56—C57—H57A	109.7
C2—Os1—O7^i^	90.49 (13)	C56—C57—H57B	109.7
C28—P2—Os2	113.79 (12)	C53—C57—C56	109.7 (3)
C42—P2—Os2	113.16 (12)	C53—C57—H57A	109.7
C42—P2—C28	103.44 (15)	C53—C57—H57B	109.7
C42—P2—C35	106.27 (16)	H57A—C57—H57B	108.2
C35—P2—Os2	118.98 (12)	C15—C14—P1	120.1 (3)
C35—P2—C28	99.30 (16)	C15—C14—C19	118.4 (3)
C7—P1—Os1	112.26 (11)	C19—C14—P1	121.2 (3)
C14—P1—Os1	114.78 (11)	C36—C37—H37	119.4
C14—P1—C7	104.90 (15)	C36—C37—C38	121.2 (4)
C14—P1—C21	104.75 (15)	C38—C37—H37	119.4
C21—P1—Os1	119.79 (11)	C22—C21—P1	121.0 (3)
C21—P1—C7	98.22 (15)	C22—C21—C26	118.2 (3)
C5—O5—Os1	122.2 (2)	C26—C21—P1	120.4 (3)
C6—O7—Os1^i^	123.3 (2)	C10—C11—H11	119.2
C6—O8—Os2^i^	123.9 (2)	C12—C11—C10	121.7 (3)
C5—O6—Os2	123.0 (2)	C12—C11—H11	119.2
O7—C6—C56	118.0 (3)	C10—C13—H13A	109.5
O8—C6—O7	124.2 (3)	C10—C13—H13B	109.5
O8—C6—C56	117.7 (3)	C10—C13—H13C	109.5
C50—C49—C58	108.9 (3)	H13A—C13—H13B	109.5
C54—C49—C50	110.1 (3)	H13A—C13—H13C	109.5
C54—C49—C58	109.3 (3)	H13B—C13—H13C	109.5
C5—C49—C50	111.2 (3)	C25—C24—C27	121.0 (3)
C5—C49—C54	111.4 (3)	C25—C24—C23	117.4 (3)
C5—C49—C58	105.9 (2)	C23—C24—C27	121.6 (3)
C50—C51—H51	109.2	C16—C15—C14	120.5 (3)
C55—C51—H51	109.2	C16—C15—H15	119.8
C55—C51—C50	109.3 (3)	C14—C15—H15	119.8
C55—C51—C52	109.7 (3)	C24—C27—H27A	109.5
C52—C51—H51	109.2	C24—C27—H27B	109.5
C52—C51—C50	110.2 (3)	C24—C27—H27C	109.5
C49—C50—H50A	109.9	H27A—C27—H27B	109.5
C49—C50—H50B	109.9	H27A—C27—H27C	109.5
C51—C50—C49	109.0 (3)	H27B—C27—H27C	109.5
C51—C50—H50A	109.9	C14—C19—H19	119.7
C51—C50—H50B	109.9	C18—C19—C14	120.6 (4)
H50A—C50—H50B	108.3	C18—C19—H19	119.7
C29—C28—P2	119.8 (3)	C16—C17—C20	120.5 (4)
C33—C28—P2	123.4 (3)	C18—C17—C16	118.3 (3)
C33—C28—C29	116.6 (3)	C18—C17—C20	121.2 (3)
C49—C54—H54A	109.7	C35—C40—H40	119.4
C49—C54—H54B	109.7	C39—C40—C35	121.3 (4)
C49—C54—C53	109.7 (3)	C39—C40—H40	119.4
H54A—C54—H54B	108.2	C44—C45—C46	118.3 (3)
C53—C54—H54A	109.7	C44—C45—C48	120.5 (4)
C53—C54—H54B	109.7	C46—C45—C48	121.2 (4)
O4—C4—Os2	178.9 (3)	C22—C23—C24	121.4 (3)
C49—C58—H58A	109.7	C22—C23—H23	119.3
C49—C58—H58B	109.7	C24—C23—H23	119.3
H58A—C58—H58B	108.2	C47—C46—H46	119.6
C56—C58—C49	109.6 (2)	C45—C46—C47	120.7 (4)
C56—C58—H58A	109.7	C45—C46—H46	119.6
C56—C58—H58B	109.7	C42—C43—H43	119.3
C7—C8—H8	119.6	C44—C43—C42	121.3 (3)
C7—C8—C9	120.7 (3)	C44—C43—H43	119.3
C9—C8—H8	119.6	C17—C20—H20A	109.5
O1—C1—Os1	178.5 (4)	C17—C20—H20B	109.5
C6—C56—C58	109.5 (2)	C17—C20—H20C	109.5
C6—C56—C55	108.8 (3)	H20A—C20—H20B	109.5
C6—C56—C57	111.3 (3)	H20A—C20—H20C	109.5
C58—C56—C55	108.1 (3)	H20B—C20—H20C	109.5
C57—C56—C58	109.2 (3)	H41A—C41—H41B	109.5
C57—C56—C55	109.9 (3)	H41A—C41—H41C	109.5
C54—C53—H53	109.5	H41B—C41—H41C	109.5
C52—C53—C54	109.7 (3)	C38—C41—H41A	109.5
C52—C53—H53	109.5	C38—C41—H41B	109.5
C52—C53—C57	109.7 (3)	C38—C41—H41C	109.5
C57—C53—C54	109.0 (3)	C25—C26—C21	121.0 (3)
C57—C53—H53	109.5	C25—C26—H26	119.5
O5—C5—O6	125.6 (3)	C21—C26—H26	119.5
O5—C5—C49	117.5 (3)	C19—C18—H18	119.5
O6—C5—C49	116.8 (3)	C17—C18—C19	121.0 (3)
C35—C36—H36	119.5	C17—C18—H18	119.5
C37—C36—H36	119.5	C40—C39—H39	119.5
C37—C36—C35	120.9 (3)	C40—C39—C38	120.9 (4)
O2—C2—Os1	177.1 (3)	C38—C39—H39	119.5
C28—C29—H29	119.0	C30—C31—C34	120.5 (4)
C28—C29—C30	122.0 (3)	C30—C31—C32	116.8 (4)
C30—C29—H29	119.0	C32—C31—C34	122.7 (4)
C47—C42—P2	122.9 (3)	C37—C38—C41	120.6 (4)
C47—C42—C43	118.2 (3)	C37—C38—C39	118.0 (4)
C43—C42—P2	118.6 (3)	C39—C38—C41	121.4 (4)
C51—C55—C56	109.8 (3)	C45—C48—H48A	109.5
C51—C55—H55A	109.7	C45—C48—H48B	109.5
C51—C55—H55B	109.7	C45—C48—H48C	109.5
C56—C55—H55A	109.7	H48A—C48—H48B	109.5
C56—C55—H55B	109.7	H48A—C48—H48C	109.5
H55A—C55—H55B	108.2	H48B—C48—H48C	109.5
C9—C10—C13	121.2 (3)	C28—C33—H33	119.4
C11—C10—C9	117.5 (3)	C28—C33—C32	121.2 (4)
C11—C10—C13	121.2 (3)	C32—C33—H33	119.4
C15—C16—H16	119.4	C31—C34—H34A	109.5
C15—C16—C17	121.2 (4)	C31—C34—H34B	109.5
C17—C16—H16	119.4	C31—C34—H34C	109.5
C51—C52—H52A	109.8	H34A—C34—H34B	109.5
C51—C52—H52B	109.8	H34A—C34—H34C	109.5
C53—C52—C51	109.6 (3)	H34B—C34—H34C	109.5
C53—C52—H52A	109.8	C31—C32—C33	121.5 (4)
C53—C52—H52B	109.8	C31—C32—H32	119.2
H52A—C52—H52B	108.2	C33—C32—H32	119.2
			
Os2—P2—C28—C29	−30.9 (3)	C29—C28—C33—C32	−1.5 (9)
Os2—P2—C28—C33	154.6 (4)	C29—C30—C31—C34	179.6 (4)
Os2—P2—C42—C47	100.1 (3)	C29—C30—C31—C32	1.1 (7)
Os2—P2—C42—C43	−72.8 (3)	C42—P2—C28—C29	−154.1 (3)
Os2—P2—C35—C36	17.1 (3)	C42—P2—C28—C33	31.4 (5)
Os2—P2—C35—C40	−171.8 (3)	C42—P2—C35—C36	146.1 (3)
Os2^i^—O8—C6—O7	−5.1 (4)	C42—P2—C35—C40	−42.7 (3)
Os2^i^—O8—C6—C56	174.87 (19)	C42—C47—C46—C45	0.8 (7)
Os2—O6—C5—O5	−1.4 (4)	C55—C51—C50—C49	61.3 (4)
Os2—O6—C5—C49	175.01 (19)	C55—C51—C52—C53	−60.5 (3)
Os1—P1—C7—C8	−140.9 (3)	C55—C56—C57—C53	58.2 (3)
Os1—P1—C7—C12	45.3 (3)	C16—C17—C18—C19	−1.6 (6)
Os1—P1—C14—C15	71.3 (3)	C52—C51—C50—C49	−59.4 (3)
Os1—P1—C14—C19	−102.8 (3)	C52—C51—C55—C56	59.4 (3)
Os1—P1—C21—C22	−1.6 (3)	C52—C53—C57—C56	−59.3 (3)
Os1—P1—C21—C26	−173.8 (2)	C47—C42—C43—C44	2.9 (6)
Os1—O5—C5—O6	18.7 (4)	C7—P1—C14—C15	−52.4 (3)
Os1—O5—C5—C49	−157.7 (2)	C7—P1—C14—C19	133.6 (3)
Os1^i^—O7—C6—O8	−12.3 (4)	C7—P1—C21—C22	120.0 (3)
Os1^i^—O7—C6—C56	167.69 (19)	C7—P1—C21—C26	−52.2 (3)
P2—C28—C29—C30	−174.7 (3)	C7—C8—C9—C10	1.1 (6)
P2—C28—C33—C32	173.2 (6)	C7—C12—C11—C10	−0.3 (6)
P2—C42—C47—C46	−176.2 (3)	C25—C24—C23—C22	−3.3 (5)
P2—C42—C43—C44	176.2 (4)	C9—C8—C7—P1	−174.2 (3)
P2—C35—C40—C39	−169.5 (3)	C9—C8—C7—C12	−0.5 (5)
P1—C7—C12—C11	174.2 (3)	C9—C10—C11—C12	0.9 (5)
P1—C14—C15—C16	−177.0 (3)	C22—C21—C26—C25	−2.6 (5)
P1—C14—C19—C18	175.6 (3)	C30—C31—C32—C33	−2.5 (10)
P1—C21—C26—C25	169.9 (3)	C35—P2—C28—C29	96.6 (3)
O7—C6—C56—C58	76.0 (3)	C35—P2—C28—C33	−77.9 (5)
O7—C6—C56—C55	−42.0 (4)	C35—P2—C42—C47	−32.3 (4)
O7—C6—C56—C57	−163.3 (3)	C35—P2—C42—C43	154.8 (3)
O8—C6—C56—C58	−104.0 (3)	C35—C36—C37—C38	0.9 (6)
O8—C6—C56—C55	138.0 (3)	C35—C40—C39—C38	0.4 (6)
O8—C6—C56—C57	16.7 (4)	C44—C45—C46—C47	2.0 (7)
C6—C56—C55—C51	179.4 (3)	C57—C56—C55—C51	−58.4 (3)
C6—C56—C57—C53	178.8 (3)	C57—C53—C52—C51	60.3 (4)
C49—C54—C53—C52	59.3 (3)	C14—P1—C7—C8	−15.7 (3)
C49—C54—C53—C57	−60.9 (4)	C14—P1—C7—C12	170.6 (3)
C49—C58—C56—C6	−178.6 (3)	C14—P1—C21—C22	−132.1 (3)
C49—C58—C56—C55	−60.2 (3)	C14—P1—C21—C26	55.7 (3)
C49—C58—C56—C57	59.3 (3)	C14—C19—C18—C17	0.8 (6)
C50—C49—C54—C53	−59.3 (3)	C37—C36—C35—P2	169.1 (3)
C50—C49—C58—C56	60.8 (3)	C37—C36—C35—C40	−2.4 (5)
C50—C49—C5—O5	−143.5 (3)	C21—P1—C7—C8	92.1 (3)
C50—C49—C5—O6	39.9 (4)	C21—P1—C7—C12	−81.7 (3)
C50—C51—C55—C56	−61.6 (4)	C21—P1—C14—C15	−155.3 (3)
C50—C51—C52—C53	60.0 (4)	C21—P1—C14—C19	30.7 (3)
C28—P2—C42—C47	−136.3 (3)	C21—C22—C23—C24	0.4 (5)
C28—P2—C42—C43	50.8 (4)	C11—C10—C9—C8	−1.3 (5)
C28—P2—C35—C36	−106.9 (3)	C13—C10—C9—C8	177.9 (4)
C28—P2—C35—C40	64.3 (3)	C13—C10—C11—C12	−178.3 (3)
C28—C29—C30—C31	0.0 (7)	C24—C25—C26—C21	−0.3 (6)
C28—C33—C32—C31	2.7 (12)	C15—C16—C17—C20	178.6 (4)
C54—C49—C50—C51	59.1 (3)	C15—C16—C17—C18	0.2 (6)
C54—C49—C58—C56	−59.5 (3)	C15—C14—C19—C18	1.4 (6)
C54—C49—C5—O5	−20.2 (4)	C27—C24—C23—C22	174.5 (3)
C54—C49—C5—O6	163.1 (3)	C19—C14—C15—C16	−2.8 (5)
C54—C53—C52—C51	−59.4 (4)	C17—C16—C15—C14	2.0 (6)
C54—C53—C57—C56	60.8 (3)	C40—C39—C38—C37	−2.0 (6)
C58—C49—C50—C51	−60.6 (3)	C40—C39—C38—C41	176.6 (4)
C58—C49—C54—C53	60.2 (3)	C45—C44—C43—C42	−0.1 (7)
C58—C49—C5—O5	98.4 (3)	C23—C22—C21—P1	−169.9 (3)
C58—C49—C5—O6	−78.2 (3)	C23—C22—C21—C26	2.5 (5)
C58—C56—C55—C51	60.6 (3)	C43—C42—C47—C46	−3.2 (6)
C58—C56—C57—C53	−60.2 (3)	C43—C44—C45—C46	−2.4 (7)
C8—C7—C12—C11	0.1 (5)	C43—C44—C45—C48	178.7 (4)
C5—C49—C50—C51	−176.9 (3)	C20—C17—C18—C19	180.0 (4)
C5—C49—C54—C53	176.8 (3)	C26—C25—C24—C27	−174.6 (4)
C5—C49—C58—C56	−179.5 (3)	C26—C25—C24—C23	3.2 (5)
C36—C35—C40—C39	1.8 (6)	C48—C45—C46—C47	−179.1 (4)
C36—C37—C38—C41	−177.3 (4)	C33—C28—C29—C30	0.1 (7)
C36—C37—C38—C39	1.4 (6)	C34—C31—C32—C33	179.1 (6)

Symmetry code: (i) −*x*+1, −*y*+1, −*z*+1.
